# How COVID-19 Has Affected Caregivers’ Burden of Patients with Dementia: An Exploratory Study Focusing on Coping Strategies and Quality of Life during the Lockdown

**DOI:** 10.3390/jcm10245953

**Published:** 2021-12-18

**Authors:** Maria Grazia Maggio, Gianluca La Rosa, Patrizia Calatozzo, Adriana Andaloro, Marilena Foti Cuzzola, Antonino Cannavò, David Militi, Alfredo Manuli, Valentina Oddo, Giovanni Pioggia, Rocco Salvatore Calabrò

**Affiliations:** 1 Department of Biomedical and Biotechnological Science, The University of Catania, 95123 Catania, Italy; mariagraziamay@gmail.com; 2 AOU Policlinico Gaetano Martino, 98125 Messina, Italy; Gialucalarosa05@gmail.com (G.L.R.); antonino.cannavo.85@gmail.com (A.C.); manulialfredo@gmail.com (A.M.); 3 Studio di Psicoterapia Relazionale e Riabilitazione Cognitiva, 98124 Messina, Italy; patrizia.calatozzo@gmail.com (P.C.); ad.andaloro@gmail.com (A.A.); marilenafoti@yahoo.it (M.F.C.); 4 Odontostomatology and Dental Surgery Study, 98124 Messina, Italy; Davidmiliti@hotmail.it; 5 Università degli Studi di Messina-Piazza Pugliatti, 1, 98122 Messina, Italy; voddo@unime.it; 6 Institute for Biomedical Research and Innovation, National Research Council of Italy (IRIB-CNR), 98164 Messina, Italy; giovanni.pioggia@irib.cnr.it; 7 IRCCS Centro Neurolesi Bonino Pulejo, 98121 Messina, Italy

**Keywords:** Alzheimer’s disease, burden, caregiver, dementia, quality of life

## Abstract

COVID-19 has caused a public and international health emergency, leading to isolation and social distancing. These restrictions have had a significant impact on the caregivers of people with dementia, increasing the burden of patient management. The purpose of this study was to investigate the stress perceived by caregivers of patients with Alzheimer’s disease (AD) during the pandemic. We used a cross-sectional survey design to evaluate the caregivers’ psychological responses and coping strategies. Eighty-four caregivers of patients with a diagnosis of AD were involved in this study by completing an online questionnaire. They presented a high perception of stress (the Perceived Stress Scale mean ± DS: 33.5 ± 4.5), and their high burden in caring was mainly related to physical difficulties (Caregiver Burden Inventory–Physical Burden mean ± DS: 15.0 ± 2.1) and perception of loss of time (Caregiver Burden Inventory–Time-dependence Burden mean ± DS: 16.5 ± 1.4). Moreover, caregivers perceived their quality of life as very low (Short Form-12 Health Survey Physical mean ± DS: 13.5 ± 2.7; Short Form-12 Health Survey Mental Health mean ± DS: 16.4 ± 4.2). Finally, we found that participants mostly used dysfunctional coping strategies, such as avoidance strategies (Coping Orientation to Problem Experiences–Avoidance Strategies mean ± DS: 39.5 ± 7.1), but these strategies did not affect the stress level of caregivers. Given that caregivers present a high burden and stress, innovative tools could be a valuable solution to investigate and support their emotional and behavioral status during difficult periods, such as the COVID-19 pandemic.

## 1. Introduction

On 30 January 2020, the World Health Organization (WHO) declared the COVID-19 pandemic an international public health emergency [1]. A few weeks after the initial outbreak in China, the total number of cases and deaths exceeded disproportionately those of the previous SARS [2,3,4,5,6]. Standard public health measures, including quarantine, social distancing, and community containment, are being used to curb the pandemic of this respiratory disease, and these new measures have changed the dynamics of social relationships, including relationships between doctors and patients, with regard to those with neuropsychiatric symptoms [6].

For these reasons, various authors have highlighted that intervention on people’s mental health is necessary given that COVID-19 has profoundly affected psychosocial status worldwide [7,8,9]. Isolation and social distancing had a significant impact on the caregiver of elderly people affected by chronic diseases, including dementia [7,8,9].

Briefly, dementia is a syndrome characterized by progressive degeneration of cognitive functions, causing impairment of normal activities and relationships in daily life [10,11,12]. Families are very important in the “long-term” management of these patients, for both therapeutic compliance and their needs [10]. Because the worsening of cognitive functions can progressively impair the ability to perform simple but essential tasks in daily life, the physical, psychological, and economic impact of dementia on individuals and their families is inevitable [13]. The “caregiver burden” consists of the emotional, physical, social, or financial burden that the caregiver feels in caring for his/her family member. It is a multidimensional concept related to the caregiver’s perception of stress while carrying out his/her care activities, and this can be influenced by psychosocial factors, such as kinship and cultural and social aspects, as well as personal characteristics, including sensitivity and vulnerability to stress [14,15]. An adequate network of services to support patients and their families is essential to reduce the burden of caregivers and delay the possible institutionalization of the patient [9]. Indeed, caregivers spend up to 10 h on daycare for the patient and meet all his/her needs, such as feeding, dressing, washing, therapy, and surveillance. A patient’s care is often complicated by behavioral problems, such as agitation, physical and verbal aggression, and disappointments. The load of the caregiver may also affect his/her work and the economic dimension, further causing emotional and psychological stress. Finally, the profound changes in the relationship between patients and caregivers may lead to feelings of frustration, despair, and anger. The coronavirus pandemic, with regard to the restrictive measures, could have made these things worse [16,17].

## 2. Materials and Methods

### 2.1. Participants and Settings

We used a cross-sectional survey design to evaluate the psychological response of caregivers of individuals with dementia during the COVID-19 pandemic lockdown, using an anonymous online questionnaire. The online survey was administered through the CAWI (Computer Assisted Web Interviewing) method: the invitation to the questionnaire was sent through the technological means offered by smartphones (i.e., WhatsApp, Facebook, Menlo Park, CA, USA) or by email. The questionnaire compilation was carried out by the online survey platform Google. The participants came from the same geographical area, i.e., the province of Messina to avoid cultural biases.

The primary caregiver was defined as the person who lives with the patient in the same home and takes primary responsibility for providing care to the patient at home.

The caregivers list has been made through the generalities and addresses provided by medical doctors (either neurologists or general practitioners) involved in the care of patients with dementia. One hundred fifty individuals were initially contacted by their clinicians, who were previously informed about the research. About 120 of them provided consent to enter the study protocol, but not all of them met the inclusion criteria. To be included in the study, caregivers had to (i) be at least 18 years of age and (ii) be the primary caregiver of a patient affected by AD.

The final sample consisted of 84 primary caregivers of patients with AD (76.2% females; mean age of years ± DS: 45.7 ± 1.3), living in the province of Messina, Italy (Table 1).

### 2.2. Procedures

Following the restrictive measures adopted by the Italian Government to deal with the pandemic, given that it was necessary to minimize face-to-face interactions and stay at home, we asked participants to fill out the online questionnaire.

They completed the questionnaires in Italian through an online survey platform (“Google Form”, Google LLC, Mountain View, CA, USA). Data collection took place from 1 April to 20 May 2020, i.e., during the first Italian lockdown.

The study complies with the principles contained in the Helsinki Declaration, and all participants provided informed consent to participate.

### 2.3. Survey Development

The questionnaire included three areas that collect closed-ended questions with evaluation on 5-point Likert scales and binary type (except for the first one that collected socio-demographic data). The survey consisted of (1) caregivers’ sociodemographic data (gender, age, education, residential position in the last 14 days, marital status, working status, type of relationship with the patient being assisted) and information about the patient’s illness, (2) psychological scales to assess the impact of the COVID-19 epidemic, and (3) tools investigating caregivers’ physical and mental health, i.e., the Perceived Stress Scale [18], the Coping Orientation to Problems Experienced-New Italian Version (COPE-NVI) [19], the Caregiver Burden Inventory (CBI) [20], and the 12-Item Short Form Survey (SF-12) [21] (Table 2).

From the psychometric perspective of scale evaluation, Cronbach’s alpha measures internal consistency across the set of individual items. Specifically, they describe the dimension of each clinical tool. In this context, we calculated Cronbach’s alpha for each dimension, except for stress level (SSP) because it consists of a single item (i.e., alpha is not available). As shown in Table 2, the items defined for the three dimensions (i.e., COPE-NVI, CBI, and SF-12) are “reliable” in capturing the characteristics of the specific dimension because they exceed the threshold of 0.70.

### 2.4. Statistical Analysis

The descriptive statistics were analyzed and expressed as mean ± standard deviation or as median ± first/third quartile for continuous variables, as appropriate; frequencies (%) were used for categorical variables. Clinical scale scores were expressed as a mean and standard deviation. The normality of the data was assessed by the Jarque-Bera test: the data met the assumption of normality.

We used linear regressions to calculate the univariate relationship between the perceived level of stress related to the caregiver burden and the scoring of the scales. All tests were two-tailed, with a significance level of *p* < 0.05. Statistical analysis was performed using SPSS Statistic 16.0 (IBM SPSS Statistics, Armonk, NY, USA).

## 3. Results

Eighty-four participants were included in the study, and all completed the online questionnaire.

As shown in Table 3, caregivers presented a high perception of stress (PSS mean ± DS: 33.5 ± 4.5). High levels of physical difficulties (CBI PH mean ± DS: 15.0 ± 2.1) and time dependence (CBI TD mean ± DS: 16.5 ± 1.4) were frequently present in the caregivers’ answers to the questionnaire. The quality of life perceived by caregivers was very low, for the aspects regarding quality of both physical and mental life (SF-12 PH mean ± DS: 13.5 ± 2.7, SF-12 MH mean ± DS: 16.4 ± 4.2). In addition, we found that participants mostly used dysfunctional coping strategies, such as avoidance strategies (COPE AS mean ± DS: 39.5 ± 7.1), with low use of functional strategies, such as orientation to the problem, positive attitude, searching for social support, and transcendent orientation.

The significant relationship between the perceived level of stress (PSS) and tools investigating caregivers’ physical and mental health are reported in Table 4. PSS was not significantly related to any dysfunctional coping strategies; thus, they did not affect the stress level of caregivers. Conversely, PSS had negative and significant relationships with the physical (SF-12 PH) and emotional (SF-12 MH) caregiver quality of life. Specifically, the worse the caregiver’s quality of life, the worse the caregiver can manage stress due to their burden, and vice versa. Finally, PSS was positively and significantly related to all the indices of high caregiver burden: time dependence (CBI-TD), development (CBI-D); physical (CBI-PH), social (CBI-SOCIAL), and emotional (CBI-EMOTIONAL). Briefly, the higher the perceived burden of the caregiver, the greater the level of stress they will face.

## 4. Discussion

As people age, there is an increase in the incidence/prevalence of chronic degenerative diseases, such as dementia, i.e., the leading cause of disability at old age [18]. Moreover, medical advances have allowed for an increase in lifespan, even in patients with chronic and disabling diseases. Consequently, the care of patients with chronic disabilities affects the quality of life of caregivers and leads to high stress with important psychosocial problems, especially during pandemics like COVID-19 [19].

The new SARS-CoV-2 pandemic and the consequent limitations have resulted in a significant deterioration in the performance of regular daily living activities, with negative effects on caregivers of patients with dementia, as observed in our sample.

The aim of this study was to investigate the stress and perceived burden of caregivers caring for patients with AD during the COVID-19 pandemic. Using univariate regression analysis, we found that participants with higher levels of perceived stress have their health severely affected. In other words, the personal health condition (both mental and physical) greatly affects the level of stress (as the health condition of the caregiver worsens, the ability to manage stress decreases). At the same time, a higher level of caregiver burden (valid for the five types of CBI explored in this study) can significantly influence the perceived stress level. Additionally, we noted that none of the dysfunctional coping strategies were able to influence the caregiver’s perceived stress level, so these strategies were not effective in this COVID-19 framework. Indeed, COVID-19 has profoundly affected the psychosocial state around the world. At an individual level, people experience fear of getting sick or dying with feelings of helplessness for both themselves and their family members [5]. Social restrictions have significantly affected the management of clinics with cancellations or postponements of outpatient visits or rehabilitation activities [7]. Considering the risk of serious COVID-19-related outcomes, most patients with dementia have been forced to stay at home. Hence, the restrictive measures may have worsened the status of patients with dementia, inducing greater discomfort and burden on the caregiver [6,7].

Some authors have shown a worsening of the neuropsychiatric symptoms of patients with dementia, such as anxiety, depression, agitation, and apathy during the COVID-19 pandemic [20,21]. In the presence of psychological and behavioral symptoms, dementia becomes more difficult and stressful to manage than other chronic conditions affecting the elderly. As a consequence, the caregivers have higher emotional and behavioral distress levels [22]. Indeed, caring for people with dementia is very challenging, and family caregivers are at higher risk for physical and mental health problems. This could be due not only to the problems related to the patient’s daily care but also to the awareness of the inexorable and uncontrollable progression of the disease [23,24]. Moreover, some studies showed that caregivers are at a greater risk of cardiovascular diseases, such as hypertension, due to the stress-related chronic inflammatory response and excessive sympathetic activation [25].

Concerning the socio-demographic data, our study has highlighted a high level of stress in caregivers, especially in women, married, employed, and, above all, in cases where the caregiver was the patient’s son. In particular, stress was perceived as a consequence of the daily needs of patients with AD (and we enrolled only caregivers of this type of dementia), such as assistance in feeding, dressing, bathing, and administering daily therapy. However, stress was higher when caregivers had to deal with neuropsychiatric disorders, such as behavioral problems, agitation, and verbal aggression. According to previous studies, perceived stress primarily affects the perception of time-wasting and physical health, as well as the quality of life. This latter was rated as very low by our sample [26,27,28,29,30].

It is noteworthy that the majority of the sample reported a worsening of stress and family care-related burden during this period, with regard to both clinical and socio-economic aspects [31,32]. In more detail, the reorganization of the healthcare system with the increase of acute wards/services to face COVID-19 and a reduction/closure of social and healthcare services for chronic illness has caused a decrease and/or interruption of the outpatient clinic and/or homecare dedicated to dementia [4]. This has caused an overload on the burden of caregivers who also had to deal with some clinical/health practices for which they did not feel properly prepared or trained [9,32,33,34,35]. Furthermore, the reduction of physical contact and social relationships did not allow caregivers to perceive adequate psychophysical and mental support, with a reduction in playful activities, increasing the PSS and worsening their quality of life [9,31,32,33,34,35]. According to recent studies, these sudden changes had an immediate impact on the caregiver’s burden by increasing the possibility of precipitating feelings of loneliness, social isolation, and increasing stress levels due to social distancing efforts [9,31,32,33,34,35].

## 5. Strengths and Limitations

The use of new technologies allowed us to administer the survey. This means of assessment is particularly useful in periods during which social distance is needed to avoid contagions, like during this terrible pandemic. As technological interventions have proven useful in the care of patients with dementia [36,37], future studies could deepen the use of telemedicine for caregivers of patients with AD as an assessing tool and psycho-emotional support for both patients and their caregivers.

The present study had some limitations. The study involved a small sample of caregivers of patients with AD, so there may be difficulties to generalize the results to the patients’ population. However, we have focused only on a specific type of dementia, so that findings by our sample might be more homogeneous, given that the different kinds of dementia often have different symptoms and disease progression.

Additionally, this study considers the self-selection issue [38]. The caregivers have voluntarily decided to participate in the questionnaire, probably due to their abilities in using technological devices. Therefore, this selection bias might have affected the accuracy of results, also due to the lack of information concerning the caregivers who were not able to fill out the online questionnaire.

Furthermore, there is no follow-up period, and it is not certain if the results obtained would have lasted over time, also considering the lack of data regarding the burden of caregivers in the pre-COVID era. Future studies are needed to compare the situation resulting from COVID-19 with others occurring out of this health emergency.

We did not collect data on the cognitive, psychological, and physical state of the AD patients, having collected the information from their caregivers, so we can only assume the presence of patient’s behavioral changes. Finally, we did not collect data on the amount of time spent by the caregivers with the patients: in the survey, the caregiver was asked to answer only if he/she was the main person in charge of the patient’s care, i.e., it was the person who spent more time with the patient than other family members. In future research, it will be necessary to extend the study to a larger sample and increase the involvement of family members and use specific assessment tools for patients as well.

## 6. Conclusions

To summarize, this study has evaluated the burden of caregivers of patients with AD during the first Italian COVID-19 lockdown. We found that there was an increase in the caregiver’s PSS with a worsening of their quality of life. We believe that innovative tools, such as online questionnaires or telemedicine, could be a valuable solution to investigate these concerns and support caregivers of people with dementia during more difficult periods, as the COVID-19 pandemic is. These aspects are fundamental to favor the correct management of chronic diseases at old age. Therefore, healthcare policies and assistance services that provide support to the crucial needs of both frail people and family members caring for them should be developed and promoted.

## Figures and Tables

**Table 1:** Descriptive analysis of patients’ and caregivers’ characteristics.

Patients	84
Age (years)	62.9 ± 4.1
Caregivers	84
Relation to patients	
Son/Daughter	54 (64.3%)
Spouse/Partner	23 (27.3%)
Other	7 (8.4%)
Age (years)	45.7 ± 9.3
Gender	20 (23.8%)
Male	64 (76.2%)
Female	
Education	15.38 ± 2.38
Professions	
Freelancer	17 (21.0%)
Employee	41 (48.0%)
Housewife	16 (19.0%)
Other	10 (12.0%)
Marital Status	
Single	35 (41.7%)
Married	42 (50.0%)
Divorced	7 (8.3%)
Sons	
Yes	45 (53.6%)
No	39 (46.4%)

Mean ± standard deviation was used to describe continuous variables; proportions (numbers and percentages) were used to describe categorical variables.

**Table 2:** Clinical assessment tools.

Test/Scale Description	Description
PSS	The Perceived Stress Scale (PSS) is the most widely used psychological instrument for measuring the perception of stress. It is a measure of the degree to which situations in one’s life are appraised as stressful. Items were designed to tap how unpredictable, uncontrollable, and overloaded respondents find their lives. The scale also includes a number of direct queries about current levels of experienced stress. The items are easy to understand, and the response alternatives are simple to grasp. The questions in the PSS ask about feelings and thoughts during the last month. Regarding the psychometric properties of PSS, it has been shown that it can be used reliably and repeatably to measure perceived stress.
COPE-NVI	The Coping Orientation to Problems Experienced is a self-report questionnaire that considers the coping strategies. The tool consists of five large, essentially independent dimensions: social support, avoidance strategies, positive attitude, problem-solving, and turning to religion. The COPE-NVI can be considered a useful and psychometrically valid tool for measuring coping styles in the Italian context.
CBI	The Caregiver Burden Inventory is a tool for the evaluation of the care load, developed for caregivers of Alzheimer’s disease and dementia patients. It is a self-report tool, compiled by the main caregiver. It is a tool for quick completion and easy understanding. Divided into 5 sections, it allows us to evaluate different stress factors: objective load, psychological load, physical load, social load, and emotional load. Regarding the psychometric properties of CBI, it has been shown to be a reliable and repeatable tool.
SF-12	The SF-12 is a self-reported outcome measure assessing the impact of health on an individual’s everyday life. It is often used as a quality of life measure. The SF-12 is a shortened version of its predecessor, the SF-36, which itself evolved from the Medical Outcomes Study. The SF-12 was created to reduce the burden of responsibility, and it has been shown that SF-12 can be used reliably and repeatably to measure the quality of life.

**Table 3:** Average of the clinical scale of caregivers.

Test/Scale	Caregivers	
	Mean ± SD	Range
COPE SS	24.2 ± 3.8	14–35
COPE AS	39.5 ± 7.1	19–58
COPE AP	29.2 ± 6.5	14–42
COPE OP	25.3 ± 4.8	14–37
COPE TO	19.4 ± 2.5	13–25
SF-12 PH	13.5 ± 2.7	8–18
SF-12 MH	16.4 ± 4.2	6–27
CBI TD	16.5 ± 1.4	0–20
CBI D	8.2 ± 6.9	0–20
CBI PH	15.0 ± 2.1	0–16
CBI SOCIAL	4.7 ± 5.1	0–19
CBI EMOTIONAL	5.1 ± 3.1	0–16
PSS	33.5 ± 4.5	3–38

Legend: Perceived Stress Scale (PSS) cut-off > 14.0; Coping Orientation to Problem Experiences (COPE) Average (DS) in Italy: Social Support (SS) 27.7(8.4), Avoidance Strategies (AS) 23.5(5.1), Positive Attitude (PA) 30.9(6), Problem Orientation (PO) 32(6.7), Transcendent Orientation (TO) 22.7(5.6); Caregiver Burden Inventory Total (TOT) cut-off > 36.0: Time-dependence Burden (TD), Developmental Burden (D), Physical Burden (PH), Social Burden (Social), Emotional Burden (Emotional); Short Form-12 Health Survey Total (SF-12 TOT) cut-off < 50; Short Form-12 Health Survey Mental Health (SF-12 MH) cut-off < 45.5; Short Form-12 Health Survey Physical (SF-12 Ph) cut-off < 50.

**Table 4:** Univariate regression models for a perceived level of stress (PSS).

Variable	Coefficient	*t*-Test	*p*-Value
Constant	16.814	3.2	0.002
COPE SS	0.278	1.3	0.197
Constant	25.334	5.4	0.000
COPE AS	−0.045	−0.38	0.702
Constant	23.747	6.2	0.000
COPE AP	−0.006	−0.05	0.960
Constant	18.727	4.24	0.000
COPE OP	0.191	1.11	0.269
Constant	33.318	5.23	0.000
COPE TO	−0.503	−1.54	0.127
Constant	37.843	9.64	0.000
SF-12 PH	−1.056 **	−3.71	0.000
Constant	44.643	19.46	0.000
SF-12 MH	−1.282 **	−9.49	0.000
Constant	19.207	15.73	0.000
CBI TD	0.509 **	4.5	0.000
Constant	19.069	16.94	0.000
CBI D	0.547 **	5.2	0.000
Constant	18.996	18.43	0.000
CBI PH	0.649 **	6	0.000
Constant	20.433	20.07	0.000
CBI SOCIAL	0.663 **	4.51	0.000
Constant	21.441	22.02	0.000
CBI EMOTIONAL	0.757 **	3.59	0.001

Significance levels of 1% (**) for coefficients by z-test are in bold. Legend: Perceived Stress Scale (PSS) cut-off > 14.0; Coping Orientation to Problem Experiences (COPE) Average (DS) in Italy: Social Support (SS) 27.7(8.4), Avoidance Strategies (AS) 23.5(5.1), Positive Attitude (PA) 30.9(6), Problem Orientation (PO) 32(6.7), Transcendent Orientation (TO) 22.7(5.6); Caregiver Burden Inventory Total (TOT) cut-off > 36.0: Time-dependence Burden (TD), Developmental Burden (D), Physical Burden (PH), Social Burden (Social), Emotional Burden (Emotional); Short Form-12 Health Survey Total (SF-12 TOT) cut-off < 50; Short Form-12 Health Survey Mental Health (SF-12 MH) cut-off < 45.5; Short Form-12 Health Survey Physical (SF-12 Ph) cut-off < 50.

## Data Availability

The data are available from the online database or on request from the corresponding author.
